# Clinical effectiveness of esophageal stricture dilation using an improved endoscopic attachment cap in adults with eosinophilic esophagitis

**DOI:** 10.1055/a-2606-7785

**Published:** 2025-06-04

**Authors:** Alain M. Schoepfer, Luc Biedermann, Andrea Kreienbuehl, Ekaterina Safroneeva, Catherine Saner, Philipp Schreiner, Thomas Greuter, Alex Straumann, Jeanine Wakim El-Khoury

**Affiliations:** 1 Division of Gastroenterology and Hepatology, Centre Hospitalier Universitaire Vaudois (CHUV) and University of Lausanne, Lausanne, Switzerland; 2 Department of Gastroenterology and Hepatology, University Hospital Zurich, Zurich, Switzerland; 3 Institute of Social and Preventive Medicine, University of Bern, Bern, Switzerland; 4 Division of Gastroenterology and Hepatology, AKH, University Hospital Vienna, Vienna, Austria; 5 Division of Gastroenterology and Hepatology, GZO Wetzikon, Zurich, Switzerland

## Abstract

**Background:**

The improved endoscopic attachment cap has the following new features: 1) more rounded tip; 2) stepwise dilation in two 1-mm increments; 3) softer plastic, ensuring better contact with the endoscope tip; and 4) improved adhesive tape. We evaluated the feasibility and effectiveness of one-time esophageal stricture dilation using the new device in adults with eosinophilic esophagitis (EoE).

**Methods:**

Patients prospectively included in the Swiss EoE cohort with esophageal strictures (diameter ≤16 mm) and stricture-related symptoms underwent dilation with the new device. Symptoms were assessed using the Eosinophilic Esophagitis Activity Index patient-reported outcomes instrument before and 2 weeks after a single dilation.

**Results:**

60 patients (median age 42 years; 75% male) were evaluated. Bougienage was successful in all patients. Median esophageal diameter increased from 12 mm (interquartile range [IQR] 11–14) to 16 mm (IQR 14–16; P < 0.001). Median symptom severity dropped from 36 points (IQR 22–62) to 0 (IQR 0–12; P < 0.001). No device became detached and no severe adverse events were reported. Post-dilation pain was recorded in 31.7% (19/60).

**Conclusions:**

Esophageal stricture dilation in adults with EoE using the improved endoscopic attachment cap was feasible, clinically effective, and environmentally friendly.

## Graphical Abstract

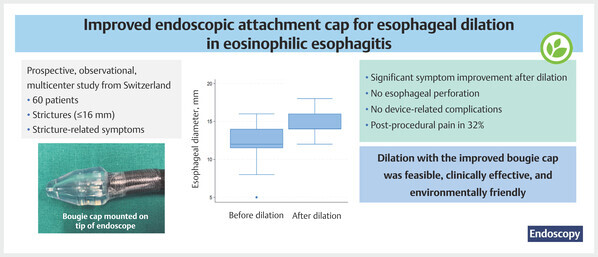



## Introduction



Eosinophilic esophagitis (EoE) is a chronic, local, immune-mediated esophageal disease, characterized clinically by symptoms related to esophageal dysfunction and histologically by eosinophil-predominant inflammation 1 2 . Adults with EoE typically present with solid food dysphagia and food bolus impaction 2 . Uncontrolled eosinophil-predominant inflammation leads to subepithelial fibrosis with consecutive esophageal stricture formation in the majority of patients 3 4 . Esophageal stricture represents a risk factor for food bolus impaction, which might be associated with esophageal perforation 2 5 . Therapeutic options include drugs (swallowed topical corticosteroids, proton pump inhibitors, dupilumab), elimination diets, and esophageal dilation in cases of stricturing disease 6 7 . In a sample of 2034 esophageal dilations in 977 patients with EoE, Dougherty et al. found that dilation was safe, with a post-procedure hospitalization rate of 0.69%, clinically significant gastrointestinal hemorrhage in 0.03%, clinically significant chest pain in 3.64%, and esophageal perforations in 0.03% per procedure 8 . The estimated esophageal perforation rate was 0.02% for bougies and 0.06% for balloons 8 .

**Fig. 1:**
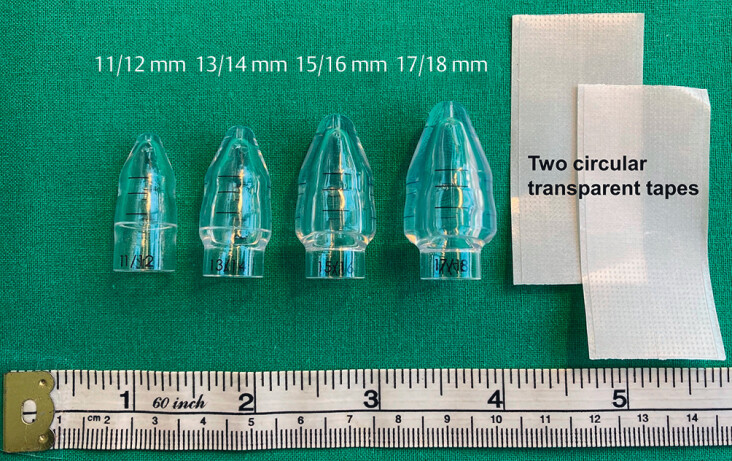
The BougieCap 2nd ed. (Ovesco Endoscopy AG, Tübingen, Germany) in diameters of 11/12, 13/14, 15/16, and 17/18 mm, and the two circular transparent tapes provided per cap for fixing the cap to the tip of the endoscope.

**Fig. 2:**
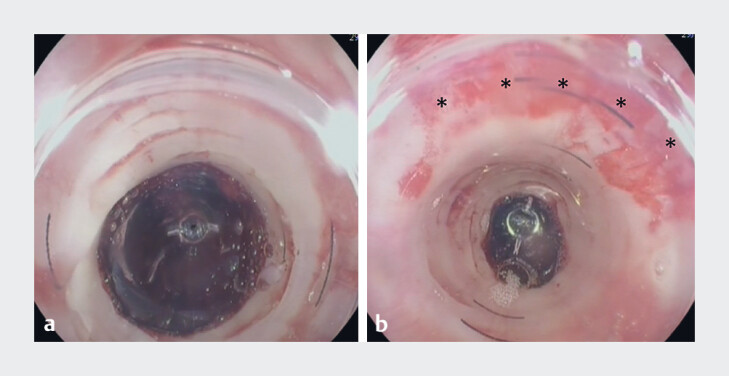
Esophageal stricture dilation using BougieCap 2nd ed. (Ovesco Endoscopy AG, Tübingen, Germany). **a** Stenosis at the gastroesophageal junction (14 mm diameter). The mucosa turns pale upon compression with the BougieCap. **b** Upon retraction, superficial laceration of the mucosa becomes apparent between the 11 and 3 o’clock positions (marked with asterisks).

Traditionally, esophageal stricture dilation in EoE is performed using either wire-guided Savary bougies or through-the-scope balloons 7 . Our group recently reported on the use of an endoscopic attachment cap (BougieCap; Ovesco Endoscopy AG, Tübingen, Germany) for esophageal stricture dilation in patients with EoE 9 . The BougieCap allows dilation of strictures of the upper gastrointestinal tract under direct visual and tactile control. The single-use, dome-shaped, transparent, hard plastic cap is attached to the scope tip using a circular tape. The first BougieCap model (1st ed.) was available in diameters of 7–16 mm. An 1.1-mm orifice at the tip of the BougieCap allowed passage of a guidewire if needed, while two lateral holes permitted insufflation of air or carbon dioxide, respectively, as well as water flushing/aspiration. Walter et al. were the first to publish their results on the efficacy and safety of benign esophageal stricture dilation using the BougieCap in 50 patients, one of whom had EoE 10 . Endoscopic bougienage was successful in 96% of patients. In our series of 50 patients with EoE, esophageal stricture dilation using this BougieCap was technically feasible, clinically effective, and safe in all patients 9 . However, our study revealed limited adhesive power of the tape, as one cap slipped and needed endoscopic retrieval. Ovesco released a second edition (2nd ed.) of the BougieCap with an improved design, characterized by several new features compared with the first design 11 . First, the new device comes with better adhesive tape that does not need cutting with scissors if length modification is needed, and the tape can be removed easily without causing harm to the plastic sheet at the tip of the endoscope ( Fig. 1 ; see also **Fig. 1s** in the online-only Supplementary material). Second, the dilation is performed in a stepwise manner in two 1-mm increments. Three clearly visible black lines allow the endoscopist to judge the diameter of an encountered stricture during the procedure. Third, the BougieCap 2nd ed. has a more rounded shape compared with the conical shape of the first edition, reducing mechanical trauma during dilation. Fourth, the softer plastic ensures better contact with the endoscope tip. The BougieCap 2nd ed. is available in diameters from 7/8 mm to 17/18mm ( Fig. 1 ). As with the first edition, the effect of dilation with BougieCap 2nd ed. is immediately visible through the appearance of a superficial laceration ( Fig. 2 ).

Green endoscopy to reduce carbon footprint is becoming an increasingly important topic. In a previous study, use of the BougieCap was associated with a 99% reduction in waste compared with conventional dilation techniques using through-the-scope balloons and Savary bougies 12 .

We evaluated the feasibility and effectiveness of one-time esophageal stricture dilation using the BougieCap 2nd ed. in 60 adults with EoE enrolled into the Swiss EoE Cohort Study (SEECS).

## Methods

### Patients and methods

Adult patients with an established EoE diagnosis are prospectively recruited into SEECS 13 . This national cohort was established in 2016 and benefits from fully electronic data capture using REDCap (Nashville, Tennessee, USA) 14 . SEECS currently follows 804 adult patients with EoE. The following inclusion criteria apply: 1) symptoms of esophageal dysfunction; 2) peak eosinophil count ≥15 per high-power field (magnification ×400); and 3) exclusion of other conditions associated with esophageal eosinophilia 1 . Patients are typically included into SEECS during a scheduled follow-up esophagogastroduodenoscopy. Before the endoscopy, patients complete (on a handheld device) the validated symptom-based Eosinophilic Esophagitis Activity Index patient-reported outcomes questionnaire (EEsAI PRO; range 0–100 points, with higher values indicating more symptoms) about symptoms in the previous 7 days 15 . The content validity, construct validity, criterion validity, feasibility, and responsiveness of the instrument have been demonstrated in several studies and randomized clinical trials 15 16 . Endoscopic activity was assessed using the validated Eosinophilic Esophagitis Endoscopic Reference Score grading system (range 0–8 points) ( **Table 1s** ) 17 . Stricture length was assessed as the length of the superficial laceration after dilation. For assessment of histologic activity, at least three biopsies were taken from both the proximal esophagus and the distal esophagus 7 . The study was approved by the six ethical committees covering both the German- and French-speaking parts of Switzerland (CER-VD 148/15).

To be eligible for dilation with BougieCap 2nd ed., patients had to have clinically active EoE, defined as EEsAI PRO ≥20 points, and have an esophageal diameter ≤16 mm, as measured using BougieCap 2nd ed. 15 . Patients were recruited by participating gastroenterologists in two tertiary referral centers. The decision to use the BougieCap 2nd ed. model to start with was at the discretion of the gastroenterologist who determined the minimal esophageal caliber after careful inspection. The minimal esophagel diameter (in mm) was defined as a caliper that was associated with resistance during passage of the BougieCap and/or a visible superficial laceration of the esophageal mucosa.

All procedures were performed under propofol sedation using either Olympus GIF-HQ190 (Olympus, Tokyo, Japan) or Pentax EG-2990I (Pentax, Tokyo, Japan) high definition video gastroscopes. Correct usage of the BougieCap 2nd ed. was demonstrated to participating gastroenterologists by the principal investigator during a zoom meeting in February 2023. Participating endoscopists followed the recently published UK guidelines for esophageal dilation 18 .

Technical feasibility, clinical efficacy, and safety were assessed after one dilation session. One week after esophageal stricture dilation, patients were contacted by phone to inquire about the presence of post-procedural thoracic pain. Two weeks after dilation, patients once again completed the EEsAI PRO questionnaire. Anti-eosinophil treatment was not changed until patients had returned the completed EEsAI PRO questionnaires. None of the patients received topical corticosteroid injections after esophageal dilation. The price for one BougieCap 2nd ed. in Switzerland was approximately 80 US dollars.

### Statistical analysis

Data were entered into an excel sheet (Microsoft excel 2010; Microsoft Corporation, Redmond, Washington, USA). The statistical analyses were performed using Stata (version 16 IC, College Station, Texas, USA). QQ-plots were used to analyze data distribution. Results of numerical data are presented either as mean (SD) for normally distributed data or median, interquartile range (IQR; reported as Quartile 1 to Quartile 3), and range for non-normal data. The chi-squared test was used to explore associations of categorical data between two groups. The Wilcoxon rank sum test was used to explore associations of non-normal numerical data between two groups. For the purposes of this study, a *P* value of <0.05 was considered to be statistically significant.

## Results

### Baseline characteristics

We recruited 67 patients with EoE, of whom 7 did not complete the EEsAI PRO questionnaire within 2 weeks after dilation and were, therefore, excluded. The baseline characteristics of the included 60 patients are shown in Table 1 . None of the patients participated in the published study evaluating the BougieCap 1st ed. 9 . Median age at inclusion was 42 years, and median disease duration from time of diagnosis was 5 years. Median symptom severity score before dilation was 36 points. Esophageal strictures that could not be passed with the standard gastroscope were found in 16.7% of patients.

**Table:** **Table 1** Demographic and disease-specific characteristics at baseline.

	n = 60
Sex, n (%)	
Male	45 (75.0)
Female	15 (25.0)
Age at BougieCap dilation, median (IQR) [range], years	42 (22–63) [18–75]
EoE disease duration since diagnosis, median (IQR) [range], years	5 (2–11) [0–12]
EoE disease duration since symptom onset, median (IQR) [range], years	10 (4–17) [1–28]
Diagnostic delay, median (IQR) [range], years	4 (1–8) [0–27]
GERD (ever diagnosed), n (%)	5 (8.3)
Endoscopically active GERD at time of BougieCap dilation	0
Treatments, n (%)	
None	6 (10.0)
STC	41 (68.3)
PPI	8 (13.3)
STC + PPI	2 (3.3)
Elimination diet	3 (5.0)
Dosage of STC, median (IQR) [range], mg	1 (0.5–2) [0.4–2]
Baseline symptom severity (EEsAI PRO score), median (IQR) [range]	36 (22–62) [20–78]
Peak eosinophil count per high power field, median (IQR) [range]	9 (0–42) [0–118]
Endoscopic activity (EREFS range 0–8), median (IQR) [range]	4 (2–7) [2–8]
Previous esophageal dilation, n (%)	35 (58.3)
Esophageal stricture ≤11 mm, n (%)	10 (16.7)
Location of esophageal stricture, n (%)	
Proximal	18 (30.0)
Distal	40 (66.7)
Proximal and distal	2 (3.3)
EEsAI PRO, Eosinophilic Esophagitis Activity Index patient-reported outcome; EoE, eosinophilic esophagitis; EREFS, Eosinophilic Esophagitis Endoscopic Reference Score; GERD, gastroesophageal reflux disease; IQR, interquartile range; PPI, proton pump inhibitor; STC, swallowed topical corticosteroid.	

### Clinical effectiveness of EoE stricture dilation using BougieCap 2nd ed.

**Fig. 3:**
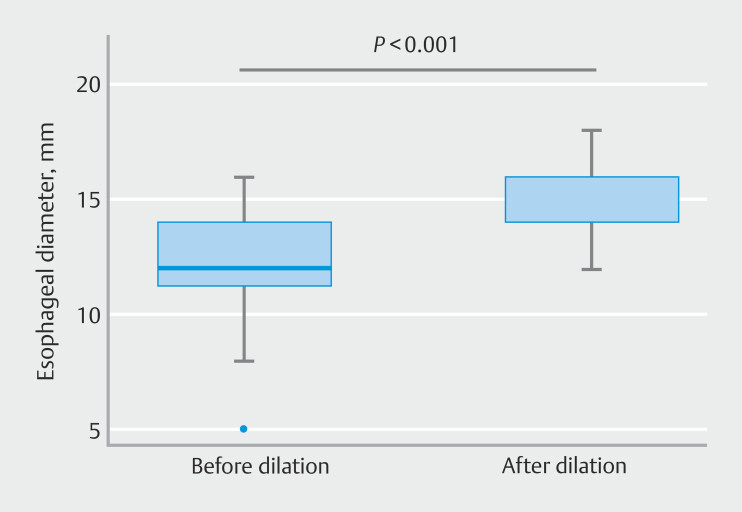
Horizontal box-plots showing esophageal diameter (in mm) before and after esophageal dilation. The box contains 50% of all values (percentile 25 to 75), the horizontal line in the box denotes the median (percentile 50). The median esophageal diameter was 12 mm (interquartile range [IQR] 11–14, range 5–16) before dilation and 16 mm (IQR 14–16, range 12–18) after dilation.

Stricture dilation was technically feasible in all patients ( Fig. 2 ). All dilations were performed without the need for a guidewire or fluoroscopic guidance. The median esophageal diameter was 12 mm before dilation and increased to 16 mm after dilation ( *P* <0.001) ( Table 2 , Fig. 3 ). A median of two BougieCaps were used (IQR 2–2, range 1–3) per dilation session ( **Table 2s** ). Stricture length was 1–2 cm in 76.6% of patients and 3–4 cm in 23.4% of patients. Superficial lacerations, interpreted as signs of successful dilation, were documented in all patients. No complications were observed with respect to need for hospitalization or esophageal perforation. One patient required hemostasis with placement of a clip in the superficial laceration. Transient thoracic pain in the days after dilation was reported by 31.7% of patients during the telephone interview 1 week after esophageal stricture dilation. No BougieCap became detached from the endoscope during the procedure.

**Table:** **Table 2** Esophageal diameter before and after dilation, and clinical efficacy and safety aspects.

Item	n = 60
Minimal esophageal diameter before dilation, median (IQR) [range], mm	12 (11–14) [5–16]
Minimal esophageal diameter after dilation, median (IQR) [range], mm	16 (14–16) [12–18]
Increase in diameter per session, median (IQR) [range], mm	3 (3–4) [2–6]
Stricture length ^1^ , n (%)	
1 cm	11 (18.3)
2 cm	35 (58.3)
3 cm	12 (20.0)
4 cm	2 (3.3)
Symptom severity 2 weeks after dilation (EEsAI PRO score), median (IQR) [range]	0 (0–12) [0–36]
Superficial laceration after dilation, n (%)	60 (100)
Esophageal perforation, n	0
Bleeding necessitating endoscopic hemostasis, n (%)	1 (1.7)
Bleeding necessitating blood transfusion, n	0
Bleeding necessitating hospitalization, n	0
Post-dilation pain, n (%)	19 (31.7)
Slipped BougieCap, n	0
EEsAI PRO, Eosinophilic Esophagitis Activity Index patient-reported outcome; IQR, interquartile range. ^1^ Measured by length of superficial laceration after dilation.	

**Fig. 4:**
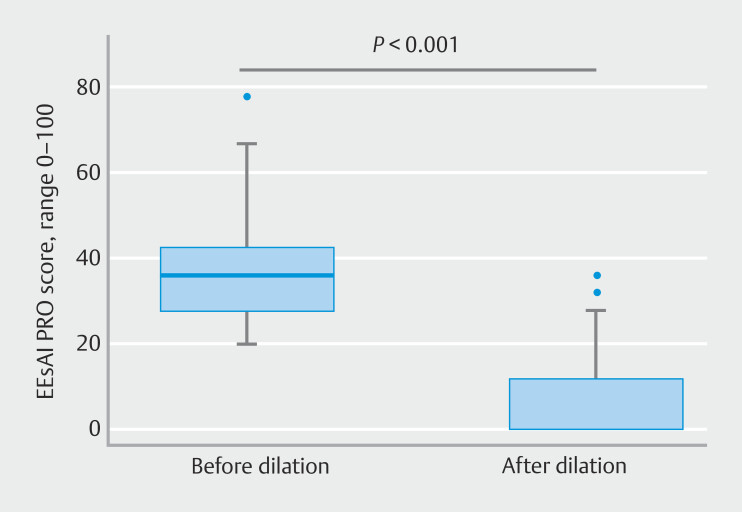
Horizontal box-plots showing symptom-based Eosinophilic Esophagitis Activity Index patient-reported outcomes (EEsAI PRO) score before and 2 weeks after esophageal dilation. The box contains 50% of all values (percentile 25 to 75), and the horizontal line in the box denotes the median (percentile 50).

EoE-related symptoms had significantly improved 2 weeks after esophageal dilation; the median EEsAI PRO score was 36 points before dilation and 0 points at 2 weeks after dilation ( *P* < 0.001) ( Fig. 4 ).

## Discussion

We present the results of the first study to systematically evaluate the feasibility and efficacy of one-time esophageal stricture dilation using the BougieCap 2nd ed. in adults with EoE. We found that esophageal stricture dilation was technically feasible and clinically effective.

Our group recently published our experiences with esophageal stricture dilation using the BougieCap 1st ed. in a cohort of 50 adults with EoE 9 . We found that dilation was technically feasible, clinically effective, and generally safe. We identified a risk of the BougieCap slipping during retraction of the endoscope, which was related to inadequate adhesion of the tape and the inflexible plastic segment of the BougieCap that is in contact with the tip of the endoscope. These shortcomings have been rectified in the design of the BougieCap 2nd ed. Dilation can be performed gradually in two 1-mm steps under direct visual control, supported by the clearly visible millimetric markings of the BougieCap 2nd ed. We did not experience a particular clinical impact of the more rounded head of the BougieCap 2nd ed. compared with the conical shape of the first edition.

In a previous study, the BougieCap was associated with less waste and a reduced carbon footprint compared with traditional methods of esophageal stricture dilation using either through-the-scope balloons or single-use bougies 12 . Green endoscopy is becoming an increasingly important topic, and various stakeholders agree that efforts should be undertaken to reduce the carbon footprint associated with gastrointestinal endoscopy 19 .

To date, there has been no published evidence on the use of the BougieCap 2nd ed. for stricture dilation in patients with EoE or in patient populations with other esophageal diseases.

Our study has several strengths as well as some limitations. We present the first study to evaluate the clinical efficacy of esophageal stricture dilation using BougieCap 2nd ed. in adults with EoE. The patient sample was sufficiently large to support our conclusions with respect to feasibility and effectiveness. However, the patient sample was too small to draw definitive conclusions regarding safety. The assessment of clinical, endoscopic, and histologic activity was performed using validated instruments that were specifically developed for adult patients with EoE. Procedures were performed by endoscopists considered to be experts in the field of EoE and esophageal stricture dilation. However, the results of our study have to be interpreted with some limitations in mind. We did not compare the effectiveness of BougieCap 2nd ed. dilation with that of other techniques of esophageal bougienage, such as through-the-scope balloons or Savary bougies. In addition, the clinical effectiveness was assessed 2 weeks after BougieCap dilation, and long-term data on the clinical improvement still need to be generated. Furthermore, most esophageal strictures dilated with BougieCap 2nd ed. were short. The clinical effectiveness and safety of dilation of strictures longer than 4 cm still need to be clarified.

In summary, the concerns associated with the BougieCap 1st ed. have been addressed in the design of BougieCap 2nd ed. As such, esophageal stricture dilation using BougieCap 2nd ed. in adults with EoE was feasible and clinically effective.

## Green stamp explained

In this study, dilation with an improved endoscopic attachment cap was shown to be feasible and clinically effective. In previous studies, this device was shown to be associated with less waste and a reduced carbon footprint compared with traditional methods of esophageal dilation with either through-the-scope balloons or single-use bougies.

## Correction: Clinical effectiveness of esophageal stricture dilation using an improved endoscopic attachment cap in adults with eosinophilic esophagitis

In the above-mentioned article the name of Jeanine Wakim El-Khoury has been corrected. This was corrected in the online version on June 18, 2025.

## Supplementary Material

Supplementary Material Zusatzmaterial
