# Correction: Clinical effectiveness of esophageal stricture dilation using an improved endoscopic attachment cap in adults with eosinophilic esophagitis

**DOI:** 10.1055/a-2637-3971

**Published:** 2025-06-18

**Authors:** Alain M. Schoepfer, Luc Biedermann, Andrea Kreienbuehl, Ekaterina Safroneeva, Catherine Saner, Philipp Schreiner, Thomas Greuter, Alex Straumann, Jeanine Wakim El-Khoury

**Affiliations:** 1 Division of Gastroenterology and Hepatology, Centre Hospitalier Universitaire Vaudois (CHUV) and University of Lausanne, Lausanne, Switzerland; 2 Department of Gastroenterology and Hepatology, University Hospital Zurich, Zurich, Switzerland; 3 Institute of Social and Preventive Medicine, University of Bern, Bern, Switzerland; 4 Division of Gastroenterology and Hepatology, AKH, University Hospital Vienna, Vienna, Austria; 5 Division of Gastroenterology and Hepatology, GZO Wetzikon, Zurich, Switzerland



In the above-mentioned article the name of Jeanine Wakim El-Khoury has been corrected. This was corrected in the online version on June 18, 2025.

