# Supplementary material for: Clinical effectiveness of esophageal stricture dilation using an improved endoscopic attachment cap in adults with eosinophilic esophagitis
Source: Endoscopy. 2025 Jun 4;57(10):1106–11. doi: 10.1055/a-2606-7785 (PMC12507039; doi:10.1055/a-2606-7785)

Thieme

Supplementary material

Clinical effectiveness of esophageal stricture dilation using BougieCap 2nd ed. in adults with eosinophilic esophagitis

Alain M. Schoepfer, Luc Biedermann, Andrea Kreienbuehl, Ekaterina Safroneeva, Catherine Saner, Philipp Schreiner, Thomas Greuter, Alex Straumann, Jeanine Wakim

Table 1s The score to assess the overall endoscopic activity based on the EREFS grading system. The overall score ranges from 0 to 8 points (0 indicates no endoscopic activity, 8 points indicates most severe endoscopic activity).

Endoscopic features	Points	Remarks
White exudates		
- Absent	0	
- Mild	1	Covering <10% of esophageal surface
- Severe	2	Covering >10% of esophageal surface
Rings		
- Absent	0	
- Mild	1	Subtle circumferential ridges
- Moderate	2	Passage of 8–9.5 mm outer diameter endoscope possible
- Severe	3	Passage of endoscope no longer possible
Edema		
- Absent	0	
- Present	1	Loss of vascular markings
Furrows		
- Absent	0	
- Present	1	
Strictures		
- Absent	0	
- Present	1	

Table 2s Number and diameters of BougieCaps used per session.

Item	Frequency
Caliber of BougieCaps used	
- 9/10 mm	7 (11.7%)
- 11/12 mm	15 (25%)
- 13/14 mm	34 (56.7%)
- 15/16 mm	34 (56.7%)
- 17/18 mm	4 (6.7%)
Number of BougieCaps used per EGD	
- 1	5 (8.3%)
- 2	53 (88.3%)
- 3	2 (3.3%)
Number of BougieCaps used per EGD, median (IQR) [range]	2 (2–2) [1–3]

Fig. 1s BougieCap 2nd ed. (17/18 mm) fixed to the tip of the endoscope using the transparent tape.

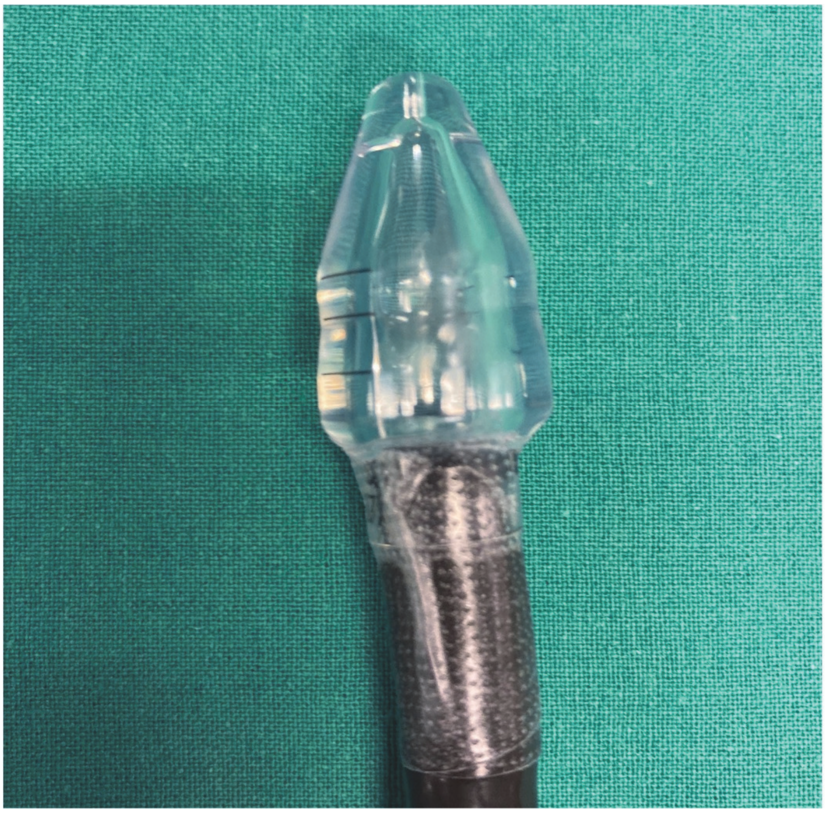

Supplement: Supplementary file 1 — Supplementary Material [file 10-1055-a-2606-7785_26082725.pdf]
